# Spiritual Well-Being and Mental Health During the COVID-19 Pandemic in Italy

**DOI:** 10.3389/fpsyt.2021.626944

**Published:** 2021-04-01

**Authors:** Ilaria Coppola, Nadia Rania, Rosa Parisi, Francesca Lagomarsino

**Affiliations:** ^1^Department of Education Sciences, School of Social Sciences, University of Genoa, Genova, Italy; ^2^Department of Economics, Management and Territory, University of Foggia, Foggia, Italy

**Keywords:** COVID-19, spiritual well-being, mental health, Italy, religious ritual, mourning process

## Abstract

During the COVID-19 pandemic in Italy, people and families experienced a new and sudden situation that forced them to stay in their homes for a long period (February 25- May 26). In this context, many people found themselves in great difficulty, not only because of the fear of contagion or the economic problems deriving from the closure of production activities but also because the virus profoundly changed the way of life in society. The “Social distancing” concept became central in all personal relationships, including close family relationships. In this situation, our paper seeks to understand the role of spirituality and religiosity in reacting to this difficult situation and in particular on the physical and psychological health of the people involved. The data we present here are part of a multidisciplinary research with a quantitative theoretical framework. As the data was collected during the first Italian lockdown, a total of 1,250 adults from all over Italy participated in the on-line questionnaire. Among the main results it emerged that the participants perceived lower levels of spiritual well-being and mental health than the pre-pandemic situation with a significant gender difference; in fact, women perceived lower mental health than men. At the same time, it is evident that spirituality and religious practices are a protective factor connected not only with psychological and mental but also physical health. Finally, it appears evident that the family is a protective factor with respect to mental health, even in a period so full of stress factors, those who did not live alone and especially those who had to take care of small children reported higher perceived mental health and a greater ability to activate coping resources.

## Introduction

The catastrophic and unstoppable nature of COVID-19 has produced a series of devastating effects from an economic, social and psychological point of view at a global level. At different times and with different strategies, the whole world is tackling the challenges dictated by the pandemic, implementing physical distancing and the partial or general closure of shops, sports centers, schools, community centers and religious institutions, also encouraging, where possible, smart-working (1). In this way, points of reference and crucial meeting places for socialization and also for the performance of religious functions, which are such necessary pillars for mutual support in general, and even more fundamental in this complex period, have been lacking. Despite this, the population, demonstrating resilience and marked adaptation skills, immediately implemented compensatory strategies to cope with the social isolation into which they were forced: in fact, innumerable initiatives were created online to offer solidarity physical, psychological and spiritual well-being. Online communities came together to practice recreational, sports and spiritual-religious activities “together.” Moreover, Yang et al., for example, argue how the use of 360° virtual tour offer a tourist experience in a moment in which the directives are to stay at home; this type of activity can, therefore, help reduce the stress caused by the pandemic (2).

These activities, particularly of a religious nature, made it possible to reduce the physical distances imposed by forced isolation without putting people's health at risk (3, 4), helping to reduce the negative effects of isolation, particularly in older people (5, 6). For many people, spiritual and religious practices give meaning, purpose and constitute a supportive community (7, 8). While, on the one hand the online initiatives managed to mitigate the negative effects of this situation, on the other, the restrictions adopted, while managing to slow down the infection, did not lead to the reduction of deaths, at least in some areas of the world where they continue to be high: today there are 1.18 million victims worldwide, of which 277.135 in Europe alone and unfortunately the statistics vary considerably day after day (9).

Although death is the natural conclusion of the life cycle, the suddenness of this event, its mass diffusion and the consequent interruption of socially shared funeral rituals and practices, on the other hand, have certainly limited the functional capacity of the victims' relatives to process death (10, 11); this has contributed to prolonged pain, thus increasing the risk of complicated bereavement (10) which can result in prolonged bereavement disorder (12). This situation has undoubtedly contributed to compromising essential areas of mental and physical functioning (13).

## Religion and Spirituality: Same Chromatic Scale?

As Thunè-Boyle et al. (14) underline, religion and spirituality appear as parts of the same chromatic scale. Although religion and spirituality represent, in fact, two different constructs, they are nevertheless strictly interconnected and difficult to separate (15). Furthermore, the literature highlights how they are the reference point for the life of many people, especially in times of difficulty (16–20). Religiosity has been defined as a multidimensional construct, oriented toward institutions and traditions (21), considered as a system of beliefs and practices (22) and defined by norms, rules, dogmas and rituals, uniting people who share the same creed (18).

Many authors have highlighted how those who are faced with adverse personal life situations such as a disease like cancer (23, 24) or the death of a loved one (25–27), particularly troubled migration paths (28, 29) or events involving the wider community such as earthquakes, tsunamis (30) tend to be more religious. As reported by Galen (31), the effects of religiosity on well-being seem to be related more to social support, a healthy lifestyle and the idea of existential certainty, than to the religious content of the beliefs in themselves; it would seem, in fact, that a large part of the advantage of religious individuals derives from being members of social groups. Spirituality, on the other hand, is seen as a more intimate dimension (18), a larger construct, an individual effort to discover the sacred or meaning of life (22) without confessional constraints (21, 32). Furthermore, it is defined by Puchalski et al. (33) as essential for humanity, a dimension, therefore, which contains within it philosophical, cultural and religious beliefs and practices.

## Spiritual Wellbeing and Mental Health

Spirituality, a source of comfort, support and meaning (7), instills the idea of a sense of belonging and existential interconnectedness, promoting mental health (34). In the literature, in fact, the accent has been placed on the association that exists between having spirituality and having a greater perception of well-being, physical and mental health (7, 19, 34, 34–37). A particularly important aspect is related to coping, or the function performed by spiritual well-being in the management of stressful events. Spiritual coping can be understood as cognitive and behavioral efforts to find or maintain meaning, purpose and connection in the face of difficult situations (38).

Furthermore, some authors over the years have argued that faith and spirituality can also be perceived as a source of resilience both from a physical, psychological and mental point of view (7, 39).

Especially in stressful situations, faith and spirituality seem to also act positively (3, 40, 41) on the immune system, particularly for older people (3, 40) who are also those most involved in religious and/or spiritual activities (15). Furthermore, spiritual well-being is defined as a state that connects the mind and body of the individual, society, intelligence and health, supporting the individual in his/her attitudes and life goals (42). According to Ellison (43), moreover, spiritual well-being includes both a psycho-social dimension and a more religious dimension, a unifying force that aims to integrate the physical, emotional and social dimensions of health. A study by Saiz et al. (44) demonstrated that spirituality in people with heart failure has broader associations with measures of psychosocial and physical symptoms than belonging to a religious organization and that religious affiliation alone did not emerge as a reliable predictor for health benefits. Indeed, it was even counterproductive: in fact they found that those who belonged to a religious organization, but with a low level of spirituality, perceived a state of anxiety and greater emotional fatigue. The literature also highlights how spiritual well-being is significantly higher in women than in men (34, 45).

Closely connected to spiritual well-being, there are spiritual needs, which include everything that refers to the need to find meaning, value in one's life, peace and a sense of connection. These needs are not necessarily exclusively religious; in fact, even those who do not have a religious faith still refer to systems of beliefs that provide feelings of meaning and purpose (46). In this period of the COVID-19 pandemic they seem to assume a role and an even deeper meaning in relation to the bewilderment that people are confronted with when faced with such a pervasive, disruptive event, creating daily fragility, fear and uncertainty. In particular, the spiritual distress in those people going through adverse situations, such as that caused by COVID-19, should not be underestimated. By spiritual distress we mean suffering connected to the impossibility of feeling meaning in life, a state of anguish that occurs when an individual experience suffering that in some way undermines their personal identity, for example by raising existential questions about the reason for that particular suffering (47, 48).

Religion and spirituality, therefore, are particularly fundamental and worthy of study in this highly complex period: COVID-19 and its physical, social and psychological consequences represent a challenge for the mental well-being of the entire world population (37, 49).

## Death in Modern Society and Its Rituals: From the Religious Dimension to Psychological Well-Being

In contemporary society characterized by a process of individualization of subjects who experience a social condition of extreme loneliness, as well as isolation, the experience of death is also progressively isolated (50) when not, tragically, removed (51, 52). There are many strategies for concealing death, some are its spectacularization and mediatic overexposure (51), others offer the solution of a “cosmic” death (52). In the latter case, the anguish of death is resolved by removing it from the individual experience. Elias (50) underlines how the loneliness of the dying person begins with their progressive isolation and has, among its consequences, the loss of the ability to empathize both with the suffering and the mourning event. In a certain sense, death is no longer part of contemporary society, which prefers to represent itself in the power of youth frozen in suspended time.

Each society has tried and tries to make sense of the human story starting from the most tragic experience of the end, which interrupts the flow of earthly life, as we know it. For many scholars, the experience of death is the central cultural theme of various societies, from ancient (53) to contemporary (54) to peasant societies in southern Italy (51). Specifically, the rituals of death give meaning to the cultural representations of the living-dead relationship (51), which are expressed through rituals organized in different practices, temporalities and aesthetics (55, 56) but all marked by the centrality of the body of the deceased and of the community committed to overcoming the mourning event.

The cultural shaping of this event, through the ritual that accompanies mourning, serves to allow the passage of the dead from the world of the living to the afterlife and, at the same time, guarantees the recovery of the community of the living (51, 57). In this perspective, the importance of the celebration of rites and of the community dimension of this celebration is clear, with a progression from the family community of loved ones (51, 55, 57) up to including the collectivity of acquaintances or an entire community. Ultimately, the death rituals that accompany mourning allow us to transcend the risk of a second and more tragic death, namely the risk for survivors of getting entangled in the mournful tragedy of the loss of a loved one and forever losing their ability to “Be stronger in the world” the closer they were to the missing person (57).

Loewenthal and Dein (58) emphasize how religious rituals offer a range of positive mental health benefits, from reduced anxiety to meaning in life and a sense of community. Moreover, as reported by Willard et al. (59), institutional religious practices sometimes affect subjective well-being even more than personal belief or individual spirituality. Finally, the lack of rituals can compromise the ability to connect with the deceased (10), strongly affecting the restoration of well-being in those who remain and who must face mourning their loss without moments of sharing with a wider relational network. which can offer support. “The absence of ritual, such as a funeral, often results in disenfranchised grief, and lacking social or cultural recognition impairs support resources that assist the grieving process” [(13). p. 80]. The process of mourning requires a complex convergence of affective responses, cognitive, behavioral, physical and spiritual adaptations that take shape through rituals and flow into an individualized equilibrium that is a source of well-being.

## The Pandemic in Italy: Images of Collective Death and Missed Rites

The pandemic has produced a shock of reality, progressively exposing us to the awareness of an extreme risk, that of death, as a collective community experience, typical of situations of war and natural catastrophes, as opposed to an individual one, in which the experience of passing away acquired the tragedy of an individual and family history.

The death curve in the representation of the disease by experts has become the daily account of the pandemic. In just 1 day, on March 28, 2020, in Lombardy alone, the most affected region of northern Italy, there were 542 deaths from COVID-19. The idea of the end took possession of us with an unprecedented emotional impact through the images from the city of Bergamo, the epicenter of one of the worst affected areas of Italy, of the dead closed in anonymous coffins, without names, without flowers, lined up in church on their last journey in solitude to the afterlife. Coffins, also in Bergamo, transported on military trucks in convoy through the streets of the deserted city, where the living were forced to stay at home, experiencing physical and spiritual distancing from loved ones who were denied the last farewell, so as to safeguard public health, eliminating the rite of the public and collective funeral that restores humanity to such a painful moment for families.

Above all, the silence and emptiness in which this convoy took place was striking. The sense of suspension of time, of loneliness, of lack of reality burst into our lives through the images that came to us from TV and social media and embody that fear, which progressively, starting from the onset of the pandemic, has turned into terror and bewilderment. The pandemic, from being a word of difficult scientific meanings, now acquires a precise, full and absolute meaning: Death.

The people who die, as well as the people who remain, relatives, family members, friends and the many spectators who feel part of an “existential community of destiny” (60), are all condemned to isolation, besides loneliness, faced with the triumph of the deadly virus. According to Migliorati (61), death in the first period of the pandemic broke out as “aseptic death,” separated from the individual experience and projected into an anonymous dimension of collective death. In the first period of lockdown, in fact, the dead and their relatives were out of the narrative. In this way, the narrative of death reduced the mournful event, albeit in its tragic nature, to a “side effect” of the pandemic, one of the many dimensions, perhaps even negligible. After all, “they are all elderly, the average age is 81 years old, they all had previous pathologies” [(61). p 40]. The most effective symbolic image of this narrative was the juxtaposition between COVID-19 and war. The pandemic was recounted as a war with its victims (especially the elderly), its heroes (especially the health personnel), its generals, ready to save us with their field strategies (the government, virologists, experts). In short, in the pandemic, death essentially broke into an interpretative scheme that saw a shift of death along the nature-culture continuum toward nature in the sign of the removal of the corpse, away from culture, capable of transcending death through ritual (57).

What happens when funerals are suspended? When the dead and their relatives go through the tragic passing alone? Because this is what happened during the lockdown in Italy: the dead were taken away alone, without a worthy accompaniment, and the living locked in their homes could not meet for worthy rituals that could ease that sudden and devastating pain and share it with people who cared. Thus, it follows that pain has no right of expression through those socially and culturally shared rituals (13); and it is precisely the absence of rituals that contributes to aggravating both the experience of mourning (13, 62) and the feelings of guilt and responsibility dictated by the conflict between what the victim's wishes were and what the state allowed (63).

The experience of death during COVID-19 goes far beyond situations of death by natural catastrophe in which the bodies are missing. In our case the relatives, reduced to members of the larger community that mourned their dead, witness the death of loved ones shut away in their homes. The tragedy of the suspension of rituals in the period of COVID-19 crossed with the tragedy of having to overcome the event alone, at home.

The pandemic has pushed the condition of loneliness, not only of the dying but also of the relatives, toward the most tragic of its epilogs. Experiments were attempted to alleviate the sense of loneliness and isolation in the face of death. For example, the creativity of a hospital chaplain in one of the most affected cities in northern Italy (Bergamo) allowed many relatives to participate remotely in the funeral ceremony via mobile phones. A coffin, a parish priest, a cell phone. All that remains of funeral participation in a period of forced isolation and media coverage of reality. As Dei (64) states “perhaps the most inhuman aspect of this experiment of suspended sociality to which we are forced, even more cruel than the dystopian imaginary fanned by those philosophers who fear our reduction to bare life, is the denial of the rituals appointed to accompany the condolences” [(64). p 2].

A cruelty that became more and more specific in the tragedy of a mourning event suspended, not overcome, entangled in the event of death, when with the passing of the weeks, toward the end of the lockdown period, from the indistinct magma of a collective death the personal stories, the family tragedies, the many faces of the dead and the remaining relatives emerged. At this point it was clear that the patriotic community which mourns its dead is unable to give meaning to the pain of the proximate community of family members, friends for the loss of a loved one.

## Aims

Given the large number of people infected and of deaths in Italy caused by the COVID-19 pandemic, this research focuses on the spiritual well-being and psychological impact of the general population during the lockdown. We believe that is important to deepen our knowledge of the perception of spiritual well-being and mental health so as to develop interventions and support people, to be ready for similar future events in order to reduce the negative consequences of a possible second wave of the virus.

We wish to pursue two main objectives. The first is to further investigate how spiritual well-being dimension was faced by Italian adults during the first weeks of lockdown subsequent to the COVID-19 outbreak. The second is to explore psychological mental health in terms of the psychological impact of the pandemic. We also set out to analyze if there were differences in the perception of spiritual well-being and mental health compared to the pre-pandemic data in the general population. The relationships between spiritual and psychological aspects were also investigated and how these two dimensions are associated with demographic variables (such as age, gender, level of education, marital status), socio-relational variables (such as people lived with, work situation, presence of children, religious beliefs) and the nearness with the COVID-19 disease, for example knowing someone who was infected or who died of coronavirus. We also assume that as spiritual well-being increases the perception of positive mental health increases and that socio-demographic variables, such as gender and age affect spiritual well-being and mental health.

## Materials and Methods

### Measure

The protocol included some questions created *ad hoc* by the research team following several focus groups, which made it possible to identify the areas to be investigated, listed below:

- Spiritual well-being: Jarel Spiritual Well-Being Scale (JSWB) (65, 66). Italian version validated by Magnano et al. (45) composed of 21 items with a five-point Likert scale from 1=strongly disagree to 5=strongly agree. The scale is composed of three factors: Faith and belief (e.g., “Prayer is an important part of my life”), Meaning of life (e.g., “I find meaning and purpose in my life”), and Quality of relationships (I am able to appreciate differences in others). The scale showed a good internal consistency (α = 0.82). The higher the scores, the greater the spiritual well-being.
- Mental health: General Health Questionnaire-12 items (GHQ-12): this scale measures the state of mental health over the past few weeks and was developed by Goldberg in the 70s and validated in Italy by Piccinelli et al. (67). The 12-item version, GHQ-12, was the most widely used (68). Participants had to report whether they experienced a particular symptom of mental distress according to a four-point Likert-type scale (“not at all”, “less than usual”, “more than usual”, “rather more than usual”). The six positive items were corrected. Participants who answered “somewhat more than usual” or “more than usual” scored 1, while those who answered “less than usual” or “not at all” scored 0 (the so-called “0-0-1-1 method”) (68). A total score ranged from 0 to 12 points; higher scores indicate worse health. The scale showed a good internal consistency (α = 0.73).
- Knowledge of people (acquaintance, friend, relative) who had contracted COVID-19 and/or someone who had died from COVID-19.
- Compilation of a socio-demographic data sheet which was included in the questionnaire. The variables taken into consideration are: age, educational qualification, marital status, current job situation, people with the subject lives, children/no children, religious belief.

### Procedure

This is a multidisciplinary research which is part of a quantitative theoretical framework. As the data was collected during the lockdown, the questionnaire was administered online. The research was proposed via a link with access to the questionnaire sent by email, WhatsApp, discussion forums and social networks such as Facebook. The platform used for the questionnaire is Microsoft Forms. Before compiling, participants read the aims of the study, the themes proposed, the type of reconstruction, the informed consent stating that participation was voluntary and that they could withdraw at any time by closing the browser window. The inclusion criteria were being at least 18 years old and living in Italy during the lockdown due to the COVID-19 pandemic disease. The convenience sample was recruited through a random cascade sampling, starting from some subjects known by the research team. The research, having an exploratory character, does not want to restore a representative image of the Italian population but rather give a picture of the perceptions of the population during the lockdown in relation to their spiritual and mental health. The compilation of the protocol lasted on average about 22 min per participant.

The data were collected in compliance with the privacy and the research ethics code of the Italian Association of Psychology, after the protocol was approved by the ethics committee of the Department of Education Sciences of the University of Genoa. The research lasted 10 days and was carried out after the first 2 weeks of lockdown. About two thirds of the questionnaires were compiled on the first day of the questionnaire launch, in line with recent and similar studies during the pandemic crisis (49, 69).

### Participants

A total of 1,250 adults from all over Italy participated in the on-line questionnaire. Most respondents were women (77.3%), young adults (age M = 42.6 years, SD = 15.7; range 19–88), married or cohabiting with partner (48.3%) or single (41.5%), without children (54.71%), living in a large center (47.9%), employed or self-employed (53.2%), and high education levels (59.8% hold at least a University degree). Participants were born mostly in Italy (96.9%), with the rest indicating 23 different countries of birth. Ecuador, Germany and Romania were the most prevalent. In **Table 2** we report the socio-demographic characteristics in detail.

With regard to religious belief, 40.9% of the participants declared that they were agnostic/atheist or did not have any religious beliefs; 57.4%, on the other hand, declared that they referred to a religious belief, of which the majority (53%) were Catholic Christians.

### Data Analysis

Descriptive statistics were calculated for sociodemographic characteristics and information about variables, consisting of frequencies and percentages, while the scores of Jarel Spiritual Well-Being Scale (JSWB) and, General Health Questionnaire (GHQ-12) were expressed as means and standard deviations. Moreover, for JSWB and GHQ-12, skewness and kurtosis values were obtained, with no further interpretation due to the large sample size (70, 71). To investigate the dichotomic variable (gender differences, have children/no children, religious belief and the closeness with the COVID-19 disease such as knowing someone who was infected or died from coronavirus) in relation to JSWB and GHQ-12, *t*-tests were used for independent samples. To compare the differences between our participants and the Italian normative sample and therefore in relation to the pre-pandemic data for JSWB (45) and GHQ-12 (79), *t*-tests were conducted for single samples. While variance analysis was used to investigate the differences between groups (age, marital status, work situation, people lived with) in relation to JSWB and GHQ-12, with *post-hoc* Tukey (for homogeneous variances) or Games-Howell (for non-homogeneous variances) between group comparisons in case of a significant overall F-value. Appropriate effect size statistics that adjust for differences in group sizes were obtained of Cohen's *d* for *t*-tests and η2 for ANOVAs. To explore the relationship between JSWB and GHQ-12 scales, correlation analyses (Pearson correlation coefficient r for GHQ-12 and JSWB and continuous variables) were conducted. We used multiple linear step way regressions to calculate the univariate associations between sociodemographic characteristics and JSWB and GHQ-12 scales. All tests were two-tailed, with a significance level of *p* < 0.05. Statistical analysis was performed using SPSS Statistic 18.0.

## Results

### Spiritual Well-Being and General Health: Descriptive Statistics

In Table 1, although not necessary given the size of the sample, the skewness and kurtosis analyses are reported, which show that there is a normal distribution of the data.

**Table 1:** Skewness and Kurtosis values.

	**M**	**SD**	**Skewness**		**Kurtosis**	
			**Value**	**SE**	**Value**	**SE**
GHQ-12	6.35	3.05	−0.116	0.072	−0.708	0.143
Jarel SWB scale (JSWB)	52.64	10.33	0.230	0.074	−0.449	0.148
Faith and belief (JSWB)	17.05	7.20	0.041	0.072	−1.127	0.143
Meaning of life (JSWB)	17.71	3.8	−0.294	0.072	−0.176	0.143
Quality of relationships (JSWB)	18.03	2.98	0.020	0.071	−0.361	0.142

Spiritual well-being during the COVID-19 pandemic in Italy, measured through the JSWB scale, revealed a sample mean score of 52.64 (SD = 10.33, see Table 1). As regards the JSWB Faith and belief dimension the data shows an average of 17.05 (SD = 7.20); with regard to the Meaning of life dimension the average is 17.71 (SD = 3.8) and finally, with regard to the Quality of relationships the average is 18.03 (SD = 2.98).

While the psychological impact, measured through the GHQ-12 scale, revealed a sample mean score of 6.35 (SD = 3.05; see Table 1). A total of 932 respondents (80.2%) showed common mental disorder, including adjustment disorders or stress reactions, therefore they were at risk of anxiety/depression (score ≥4), while 230 (19.8%) reported a low psychological impact (score <4).

Regarding the perceived level of spiritual well-being a significant difference between those who participated in the research (M = 52.64, SD = 10.33) and the normative sample emerged (M = 55.03, SD = 9.38) (*t*(1095) = −7.65, *p* < 0.001, Cohen's *d* = 0.24) (45) (see Table 2). Also, with regard to mental health from a comparison with the normative sample (M = 1.80, SD = 2.3) (72) a significant difference emerges from the average obtained by the participants in the research (M = 6.35, SD = 3.05, *t*(1161) = 50.90, *p* < 0.001, Cohen's *d* = 1.68) (see Table 2).

**Table 2:** Spiritual Well-Being Scale (SWBS) and General Health Questionnaire-12 (GHQ-12) comparison between participants and Italian normative sample.

**SWBS**					**GHQ-12**				
**Participants**	**Italian normative sample**	**t (df)**	***P***	**Cohen's *d***	**Participants**	**Italian normative sample**	**t (df)**	***P***	**Cohen's *d***
M (SD)	M (SD)				M (SD)	M (SD)			
52.64 (10.33)	55.03 (9.38)	−7.65 (1, 095)	0.000	0.24	6.35 (3.05)	1.8 (2.3)	50.90 (1,161)	0.000	1.68

### Spiritual Well-Being and General Health: Demographics Variables

Table 3 shows the descriptive data for all demographic variables as well as the associations between such variables and Spiritual well-being and General Health. Women showed significantly higher levels in both variables.

**Table 3:** Descriptive data for all demographic variables, associations between variables and Spiritual well-being and General Health.

**Demographic variables**	***N***	**%**	**JSWB**				**GHQ-12**			
			**M (SD)**	**t/F**	***p***	**Cohen's *d*/η${\;}_{\text{p}}^{2}$**	**M (SD)**	**t/F**	***P***	**Cohen's *d*/η${\;}_{\text{p}}^{2}$**
**Gender**										
Male	278	22.70	51.35 (10.81)	−2.21	0.03	0.16	6.01 (3.07)	−2.08	0.038	0.14
Female	944	77.30	53.01 (10.17)				6.45 (3.04)			
**Age**										
18-24	192	15.60	49.94 (9.80)	8.9	0.000	0.040	7.35 (3.05)	6.20	0.000	0.026
25-34	295	24.00	51.03 (10.05)				6.38 (3.11)			
35-44	154	12.50	51.42 (9.55)				6.30 (2.97)			
45-54	233	18.90	55.64 (9.82)				5.73 (3.08)			
55-64	265	21.50	54.00 (10.25)				6.11 (2.96)			
≥65	91	7.40	52.79 (11.34)				6.37 (2.74)			
**Educational qualification**										
Juniorhigh school	31	2.50	51.04	1.33	0.27		7.41 (3.38)	1.29	0.278	
Secondary school	466	37.60	52.48				6.39 (3.00)			
Graduation	544	43.90	52.39				6.30 (3.05)			
Postgraduate specialization	197	15.90	54.01				6.21 (3.09)			
**Marital status**										
Unmarried/Single	515	41.50	51.55 (10.64)	4.01	0.007	0.010	6.85 (3.04)	8.00	0.000	0.020
Married/Cohabiting	600	48.30	53.66 (10.11)				6.00 (2.97)			
Separated/Divorced	101	8.10	53.18 (8.44)				6.07 (3.39)			
Widower	26	2.10	50.13 (11.46)				5.33 (2.04)			

Through the test for independent samples it emerged that there is a significant difference both as regards the Spiritual Well-Being Scale and as regards the GHQ-12 scale in relation to gender: in particular, as shown in the Table 3, it emerged that on average women perceive a higher level of spiritual well-being and a lower level of mental health than men.

Moreover, through the univariate ANOVA it emerged that both as regards the JSWB scale (*F*(5, 1078) = 8.89, *p* < 0.01, $\eta_{\text{p}}^{2}$ = 0.040) and as regards the GHQ-12 scale (*F*(5, 1144) = 6.20, *p* < 0.01, $\eta_{\text{p}}^{2}$ = 0.026) there is a significant difference in relation to age; in fact, compared to the JSWB scale, *post-hoc* testing revealed significant differences between those aged 18–24 (M = 49.94, DS = 9.80) and 25–34 (M = 51.03, SD = 10.05) and those aged 45-54 (M = 55.64, DS = 9.82) and 55–64 (M = 54.00, DS = 10.25), who have a higher level of spiritual well-being than those who are aged 18–24 years. These findings indicated that younger participants have lower levels of spiritual well-being; also as regards the GHQ-12 scale, those aged between 18 and 24 (M = 7.35, SD = 3.05) have a statistically significant different level of perceived mental health compared to the other age groups (25–34, 35–44, 45–54, 55–64). These findings indicated that younger participants have a lower level of perceived mental health compared to the older participants.

Regarding the educational qualification variable, no significant differences emerged either in relation to spiritual well-being or in relation to mental health.

Regarding marital status, significant differences emerged both in the JSWB scale *F*(3, 1090) = 4.01, *p* < 0.01, $\eta_{\text{p}}^{2}=$ 0.010, and in the GHQ-12 scale (*F*(3, 1157) = 8.00, *p* < 0.01, $\eta_{\text{p}}^{2}=$ 0.020). Regarding the JSWB scale, *post-hoc* testing revealed significant differences between the unmarried (M = 51.55, SD = 10.64) and married or cohabiting (M = 53.65, SD = 10.11). These findings indicate that singles have a lower level of spiritual well-being than married or cohabiting participants. Also, for GHQ-12, *post-hoc* testing revealed significant differences between the unmarried (M = 6.85, SD = 3.04) and married or cohabiting participants (M = 6.01, SD = 2.97). These findings indicate that singles have a worse mental health than married participants.

### Spiritual Well-Being and General Health: Socio-Relational Variables

In Table 4 we report the data for some relational variables and the two scales of Spiritual well-being and General Health.

**Table 4:** Descriptive data for Socio-relational variables and associations between variables and Spiritual well-being and General Health.

**Socio-relational variables**	***N***	**%**	**JSWB**				**GHQ-12**			
			**M (SD)**	**t/F**	***p***	**Cohen's *d/η*${\;}_{\text{p}}^{2}$**	**M (SD)**	**t/F**	***P***	**Cohen's *d*/η${\;}_{\text{p}}^{2}$**
**People with the subject lives**										
Alone	142	11.50	53.67 (10.85)	3.86	0.009	0.011	5.87 (3.05)	2.21	0.085	
In the family (parents, children, brothers/sisters	705	57.10	53.09 (10.45)				6.50 (3.13)			
with partner	312	25.30	52.15 (9.79)				6.13 (2.82)			
With friends/roomates	76	6.20	49.04 (9.86)				6.55 (3.08)			
**Current job situation**										
Unchanged from before	169	22.40	53.46 (10.06)	8.38	0.000	0.025	6.00 (2.90)	0.07	0.936	
Smart working	430	57.00	54.39 (10.02)				6.10 (3.08)			
Lost job/layoffs	156	20.00	50.36 (9.55)				6.11 (3.23)			
**Children**										
No children	677	54.70	51.50 (10.28)	−4.34	0.000	0.27	6.60 (3.03)	2.99	0.003	0.18
Children	561	45.30	54.22 (10.20)				6.06 (3.05)			
**Religious belief**										
Atheists/agnostics/no beliefs	502	40.90	45.70 (7.56)	−24.24	0.000	1.46	6.23 (3.16)	−1.11	0.268	
Believers	724	57.40	57.85 (8.95)				6.43 (2.97)			

In relation to the variable *people with the subject lives F*(3, 1083) =3.86, *p* < 0.01, $\eta_{\text{p}}^{2}=$ 0.011, and work situation (*F*(2, 658) =8.38, *p* < 0.01, $\eta_{\text{p}}^{2}=$ 0.025) significant differences emerge in the JSWB scale alone. *Post-hoc* testing revealed significant differences between those who live with friends and roommates (M = 49.04, SD = 9.86) and those who live alone (M = 53.67, SD = 10.85) or with families (M = 53.09, SD = 10.45). These findings indicate that those who live with friends or roommates have a lower level of spiritual well-being than those who live alone or in families. Instead, with regard to the work situation variable during the lockdown, *post-hoc* analysis showed that there is a significant difference between those who have jobs or are on layoffs (M = 50.36, SD = 9.55) and those in an unchanged work situation compared to before (M = 53.46, SD = 10.06) or working in smart working (M = 53.39, SD = 10.02). These findings indicate that those who lost their jobs or were laid off perceived a lower level of spiritual well-being than those in unchanged work situations or in smart working.

Conversely, there is no significant difference between the work situations and the perceived level of mental health.

Moreover, with regard to the children/no children variable, those who do not have children declared a perceived worse mental health (GHQ-12) (M = 6.60, SD = 3.03) compared to those with children (M = 6.06, SD = 3.05) (*t*(1, 156) = 3, *p* < 0.01, Cohen's *d* = 0.27).

Finally, with regard to the religious belief variable, the analysis carried out shows that between those who declared themselves as atheists/agnostics or with no religious belief and those who declared a religious belief there is no significant difference in the level of perceived mental health, while there is a significant difference with respect to the perceived level of spiritual well-being (those who declared themselves as atheists/agnostics or with no religious belief = M = 45.70, SD = 7.56, score lower than those who declared a religious belief = M = 57.85, SD = 8.95, *t*(1, 074) = −24.24, *p* < 0.001, Cohen's *d*= 1.46).

### Spiritual Well-Being and General Health: Closeness to COVID-19

The closeness with the COVID-19 disease variable is presented in Table 5.

**Table 5:** Associations between “No contact with COVID-19- contact with COVID-19” and Spiritual well-being and General Health.

**Variable**	***N***	**%**	**JSWB**				**GHQ-12**			
			**M (SD)**	**T**	***p***	**Cohen's *d***	**M (SD)**	**t**	***P***	**Cohen's *d***
No contact with COVID-19	492	39.7	51.86 (10.66)	−2.190	0.03	0.18	6.43 (2.96)	0.576	0.565	
Contact with death	233	18.8	53.81 (10.42)				6.28 (3.21)			

It was verified that there is a significant difference between people who know someone who died from COVID-19 (18.8%) and who had no contact with COVID-19 (39.7%): in fact the results show that those who know people who died from COVID-19 perceive higher levels of spiritual well-being compared to those who have not had contact with COVID-19 (M = 53.81, SD = 10.42, M = 51.86, SD = 10.66, *t*(631) = −2.19, *p* < 0.05). It emerged, however, that in relation to this variable there are no significant differences as regards the GHQ-12 scale.

### Spiritual-Well-Being Scale: Faith and Belief, Meaning of Life, Quality of Relationships

Further analyzes were made to understand how the sub-dimensions of Jarel Spiritual Well-Being affect demographic and socio-relational variables differently. Tables 6, 7 show only the significant analyzes in relation to these variables.

**Table 6:** Descriptive data for Demographic variables and associations between variables and Spiritual well-being dimension: Faith and belief, Meaning of life, Quality of relationships.

**Demographic variables**	***N***	**%**	**JSWB**				**JSWB**			
			**Faith and belief**				**Meaning of life**			
			**M (SD)**	**t/F**	***p***	**Cohen's *d*/ η${\;}_{\text{p}}^{2}$**	**M (SD)**	**t/F**	***P***	**Cohen's *d*/ η${\;}_{\text{p}}^{2}$**
**Gender**										
Male	278	22.70	16.08 (7.43)	−2.52	0.01	0.18				
Female	944	77.30	17.35 (7.09)							
**Age**										
18-24	192	15.60	15.51 (6.73)	8.20	0.000	0.35	16.76 (3.68)	6.31	0.000	0.027
25-34	295	24.00	16.05 (6.95)				17.10 (3.87)			
35-44	154	12.50	16.22 (6.95)				17.76 (3.60)			
45-54	233	18.90	19.01 (6.87)				18.45 (3.68)			
55-64	265	21.50	18.21 (7.44)				18.10 (3.66)			
≥65	91	7.40	16.10 (8.15)				17.65 (3.76)			
**Marital status**										
Unmarried/Single	515	41.50	16.46 (6.97)	3.07	0.027	0.008	17.20 (3.98)	5.27	0.003	0.013
Married/Cohabiting	600	48.30	17.69 (7.3)				18.04 (3.51)			
Separated/Divorced	101	8.10	16.72 (7.31)				18.33 (4.18)			
Widower	26	2.10	15.39 (8.06)				17.80 (3.84)			

*The table shows only the significant analyzes in relation to the variables considered*.

**Table 7:** Descriptive data for Socio relational variables and associations between variables and Spiritual well-being dimension: Faith and belief, Meaning of life, Quality of relationships.

**Socio-relational variables**	***N***	**%**	**JSWB**				**JSWB**				**JSWB**			
			**Faith and belief**				**Meaning of life**				**Quality of relationships**			
			**M (SD)**	**t/F**	***p***	**Cohen's *d/η*${\;}_{\text{p}}^{2}$**	**M (SD)**	**t/F**	***p***	**Cohen's *d*/η${\;}_{\text{p}}^{2}$**	**M (SD)**	**t/F**	***p***	**Cohen's *d*/η${\;}_{\text{p}}^{2}$**
**People with the subject lives**														
Alone	142	11.5					18.19 (4.03)	4.57	0.003	0.012				
In the family (parents, children, brothers/sisters)	705	57.1					17.70 (3.79)							
with partner	312	25.3					17.87 (3.72)							
With friends//roomates	76	6.2					16.24 (3.64)							
**Current job situation**														
Unchanged from before	169	22.4	17.73 (7.08)	4.94	0.007	0.014								
Smart working	430	57	17.87 (7.07)											
Lost job/layoffs	156	20	15.80 (6.78)											
**Children**														
No children	677	54.7	16.2 (6.9)	−4.63	0.000	0.27	17.31 (3.93)	−4.09	0.000	0.24				
Children	561	45.3	18.17 (7.43)				18.22 (3.57)							
**Religious belief**														
Atheists/agnostics/no beliefs	502	40.9	10.95 (4.81)	−36.56	0.000	2.16	17.07 (3.78)	−4.91	0.000	0.29	17.83 (2.93)	−2.01	0.04	0.12
Believers	724	57.4	21.58 (5.02)				18.17 (3.76)				18.18 (2.96)			

*The table shows only the significant analyzes in relation to the variables considered*.

The Table 6 shows how there is a significant difference in relation to gender regarding the dimension Faith and belief (*t*(1142) = −2.52, *p* < 0.05, Cohen's *d* = 0.018): women perceive greater Faith and belief than men. Moreover, the table shows that both as regards the Faith and Belief dimension (*F*(5, 1147) = 8.20, *p* < 0.001, $\eta_{\text{p}}^{2}$ = 0.035) and as regards the Meaning of life (*F*(5, 1148) = 6.31, *p* < 0.001, $\eta_{\text{p}}^{2}$ =0.027) there is a significant difference in relation to age; in fact, compared to the Faith and Belief dimension, *post-hoc* testing revealed significant differences between those aged 18-24, 25-34 and those aged 45-54, 55-64, who have a higher level of Faith and belief and Meaning of life than those who are aged 18-24 and 25-34 years. These findings indicated that younger participants have lower levels of Faith and belief and Meaning of life.

Finally, with regard to the marital status, the results highlight how there is a significant difference both as regards the Faith and belief dimension (*F*(3, 1159) = 3.07, *p* < 0.05, $\eta_{\text{p}}^{2}$ = 0.008), and the Meaning of life dimension (*F*(3, 1161) = 5.27, *p* < 0.01, $\eta_{\text{p}}^{2}$ = 0.013): in particular from the *post-hoc* it emerges how those who are unmarried/single perceive a lower level of Faith and belief and Meaning of life than those who are married/cohabiting.

With regard to the socio-relational variables, the Table 7 shows that as regards the variable *people with the subject lives*, significant differences emerge in Meaning of life (*F*(3, 1154) = 4.57, *p* < 0.01, $\eta_{\text{p}}^{2}$ = 0.012). As for the Meaning of life dimension, *post-hoc* show how those who live with friends and roommates perceive lower levels of Meaning of life than those who live alone, with family or with a partner.

With regard to the Current job situation variable, as reported in Table 7, a significant difference emerges in Faith and belief dimension (*F*(3, 706) = 4.94, *p* < 0.01, $\eta_{\text{p}}^{2}$ = 0.014). From the *post-hoc* it emerges that those who have declared that their work situation has remained unchanged or have switched to smart-working perceive a higher level of Faith and belief than those who have lost their jobs or are on layoffs.

Regarding the variable children (no children/children) significant differences emerge both in the dimension of Faith and belief (*t*(1159) = −4.63, *p* < 0.001, Cohen's *d* = 0.27) and the dimension of Meaning of life (*t*(1160) = −4.053, *p* < 0.001, Cohen's *d* = 0.24): those with children report higher levels of Faith and belief and Meaning of life than those who do not. Finally, with regard to the religious belief variable, from the *t*-test it emerges, as reported in Table 7, that there is a significant difference between those who declared to have a religious belief and those who do not with respect to all three dimensions: Faith and belief (*t*(1158) = −36.33, *p* < 0.001, Cohen's *d* = 2.16), Meaning of life (*t*(1156) = −4.91, *p* < 0.001, Cohen's *d* = 0.29) and Quality of relationships (*t*(1168) = −2.02, *p* < 0.05, Cohen's *d* = 0.12): those who have declared to have a religious belief perceive greater levels of Faith and belief, Meaning of life and Quality of relationships than those who have declared not to have religious belief.

### Association Between Variables, Correlations and Regression

Pearson's correlational analyses revealed that during the lockdown there was a positive correlation between the perception of spiritual well-being and the age of the participants: with increasing age of the participants, the perceived spiritual well-being increased (*r* = 0.153, *p* = 0.01). Furthermore the psychological impact of the COVID-19 crisis has had a negative impact as regards mental health especially on younger people (*r* =-0.106, *p* = 0.01): in fact there is a negative relationship between the level of perceived mental health and the age of the participants: the risk of anxiety and stress decreases with increasing age. There is also a significant relationship between the GHQ-12 and the JSWB scale (*r* = −0.28, *p* < 0.05) and also in two of the three sub-dimensions, the Meaning of life (*r* = −0.17, *p* < 0.05) and Quality of relationships (*r* = −0.19, *p* < 0.05) in particular, there is a negative correlation between GHQ-12 and spiritual well-being: as perceived spiritual well-being decreases, perceived mental health malaise increases perceived by the participants, who are more at risk of anxiety and depression.

Further investigation highlighted the factors affecting Jarel Spiritual well-being and general health scale. The stepwise model selection in multiple linear regression analysis that considered JSWB scale as a dependent variable is presented in Table 8.

**Table 8:** Regression model: Jarel spiritual well-being scale (JSWB) as dependent variable.

Variables^*^	**B**	**SE**	**Beta**	**t**	***R*^**2**^Adj**
GHQ-12	−0.621	0.102	−0.186	−6.062	0.061
Age	0.097	0.020	0.147	4.766	
Gender	2.346	0.739	0.098	3.175	

* *There are no excluded variables in the model*.

The model has an *R*^2^ = 0.062, which means that 6% of the variance in JSWB scale is explained by the model. The *R*^2^-value was statistically significant. Mental health seems to be the biggest predictor (ß = −0.621, *p* < 0.01). While age (ß = 0.097, *p* < 0.01) and gender (ß = 2.346, *p* < 0.01) are moderate predictors.

Table 9 presents the stepwise model selection in multiple linear regression analysis, in which GHQ-12 was used as a dependent variable.

**Table 9:** Regression model: General Health (GHQ-12) as dependent variable.

Variables^*^	**B**	**SE**	**Beta**	**t**	***R*^**2**^Adj**
JSWB	−0.059	0.009	−0.198	−6.416	0.044
Gender	0.706	0.222	0.098	3.176	

* *In the model the variable age is excluded*.

The model has an *R*^2^ = 0.04, which means that 4% of the variance in mental health is explained by the model. The *R*^2^-value was statistically significant. The JSWB scale seems to be the biggest predictor (ß = −0.059, *p* < 0.001). While gender (ß = 0.23, *p* < 0.001) is a moderate predictor.

## Discussion

This research, carried out during the lockdown period in Italy, has highlighted, in accordance with research carried out before the pandemic emergency event due to COVID-19 (19, 34, 34–36, 41) how there is a connection between perceived spiritual well-being and mental and psychological health. Furthermore, the research by González Sanguino et al. (73) has shown how spiritual well-being has been found to be a protective factor for depression and anxiety.

The data presented here show how our participants perceive both lower levels of spiritual well-being (45) and lower perception of positive mental health (72, 74) compared to the situation existing pre-pandemic. Furthermore, the period of the first lockdown in Italy seems to have had more significant effects on the mental health of the population than in other countries: Italians, in fact, record lower levels of mental health compared to those that emerged in a research conducted in the same period in the United Kingdom, where generalized lockdown was not in effect (75). However, these results are in line with previous research (76) which highlights how experiencing situations of crisis and bereavement can lead to the perception of poor spiritual well-being and greater psychological distress.

With regard to demographic variables, in relation to gender, it emerges that women perceive higher levels of spiritual well-being, in line with previous research (34, 45). In particular, women perceive greater Faith and belief than men. According to some authors, the higher scores of women's spiritual well-being could be linked to the fact that women have different experiences and coping strategies to men and also to the fact that religious norms and beliefs are more compatible with roles, characteristics and behaviors socially attributed to women (34, 77, 78). In relation, instead, to mental health, women show a lower perceived level of mental health, which confirms a constant of the previous literature (79–81) and also relates to the period COVID-19 (49, 82–84).

With regard to the age variable, the data showed that the younger participants (18–24 years old) experienced a lower level of spiritual well-being, in particular Faith and belief and Meaning of life than people belonging to other age groups. In fact, from the literature it emerges that elderly people in particular benefit from religious practices and spiritual activities that positively affect their general well-being (3, 41).

Also, with regard to the perception of mental health, the data showed that the participants between 18 and 24 years old, the youngest participants, were the ones who were most affected by the lockdown situation, as also found in recent literature linked to the pandemic (85, 86). This condition of malaise could be due to the fact that young people suffered more from the lockdown restrictions because they are more used to living outside the home environment and according to González-Sanguino et al. (73) this could also be due to the fact that younger participants have fewer resources and strategies to deal with crisis situations.

With regard to the marital status variable, the research also shows that singles have a lower level of spiritual well-being (in particular Faith and belief and Meaning of life) and mental health than those who are married or living together. This may be dictated by the fact that living as a couple can be considered a source of support in a lockdown situation in which social relations are limited to mere electronic contact (87, 88).

Analyzing the socio-relational variables, it emerged that those who live with friends and/or roommates perceive a lower level of spiritual well-being and in particular Meaning of life, than those who live alone or with family. This could also be due to age, a variable that was found to be negatively correlated with spiritual well-being; in fact, those who lived with friends or roommates were university students. Still in relation to the variable people with the subject lives, as regards mental health no significant differences emerged; this may be due to the fact that compared to the pre-pandemic period all the participants, regardless of the people they lived with during the lockdown, were significantly affected by the situation.

Instead, in relation to the current job situation, it emerged that those who lost their jobs or had been laid off perceived lower levels of spiritual well-being, and in particular of Faith and belief, than those whose work situation had remained the same as before the lockdown or had switched to smart working. This is in line with that strand of literature which argues that people living in crisis situations perceive a low level of spiritual well-being (76). The fact that a difference with respect to mental health does not emerge may be due to the fact that the pandemic emergency situation led the participants to share the same destiny as regards their state of health, in which the working variable did not assume a significant weight at least in the initial stages of the emergency; an aspect that now, on the other hand, appears decidedly more salient and that the ever-increasing riots and protests in various countries are highlighting as a problematic condition.

Furthermore, with respect to the children/no children variable, it emerged that those who do not have children perceive lower levels of spiritual well-being, in particular Faith and belief and Meaning of life than those who have children, this may be due to the fact that those who have children rely more on faith and spirituality. Carter (89) argues that for many families being involved in the congregation and their own spirituality are a source of strength, support and social support in the path of life together. Indeed, in the literature it has been found that the majority of families in the world, both within and beyond religious belief systems, use different forms of expression to satisfy their spiritual needs, particularly when facing adversity (20). Although spirituality and religion are two different constructs, literature shows strong correlations and overlaps; therefore, especially considering the high percentage of believers in our sample, it is evident that religion and church attendance in Italy, despite having decreased from the 1960s to today (90) still has a significant role and is probably even more fundamental for coping with this pandemic period (30), which may be more evident in families with children. Furthermore, the data also showed that those who do not have children have perceived lower levels of mental health than those who have.

This could be due to the fact that having to take care of a child leads to an increase in coping and resilience strategies, which positively affect psychological well-being (91). Furthermore, even though families faced numerous stresses during the lockdown (92, 93) they still reported a perception of greater mental health than singles who had to go through this period alone, in quarantine at home, inevitably perceiving less material and emotional support.

From the analysis of the results, it also emerged that those who declared they had a religious belief perceived a higher level of spiritual well-being and reports higher levels in all its dimensions (Faith and belief, Meaning of life and Quality of relationships) than those who declared that they were atheist/agnostic or did not have a belief. This can be explained by the fact that, as previously seen, religion and spirituality are two closely related constructs (16–18, 22) thus making it very likely that those who reported a religious belief benefited most in terms of perception of spiritual well-being. However, our data does not reveal a relationship between religious belief and mental health.

This finding is not confirmed in the research carried out in this pandemic situation by Pirutinsky et al. (37), who found that positive religious coping, intrinsic religiosity and trust in God were strongly correlated with less stress and more positive impact; Bentzen again (30), argues how people use religion to cope with the emotional stress caused by COVID-19, arguing how religiosity increases in response to unpredictable natural disasters, such as the COVID-19 crisis.

With respect to proximity to death, the data showed that those who know people who died from COVID-19 perceive higher levels of spiritual well-being precisely because, as also emerged in the literature (3, 7, 26), spirituality is considered a possible factor of resilience, which by positively affecting mental health, is crucial for those who are going through a grieving process, and who are also, in a similar situation, deprived of the possibility of implementing the typical rituals, such as the holding of funerals that were banned during the lockdown period, and which allowed people to accompany the deceased to burial, surrounded by the affection of friends and family and which contributed to the processing of the loss and mourning itself (13, 63).

Finally, just as also found in the literature (3, 30, 34, 35, 41) the data showed that there is a relationship between spiritual well-being and mental health: from the analysis, in fact, it emerged that those who reported a lower level of spiritual well-being perceived a worse level of mental health. Finally, with regard to spiritual well-being, the data showed that mental health is the major predictor, while gender and age are moderate predictors. In fact, as also seen from the data reported previously and from the literature, age (3) and gender (34) have a significant impact on the perception of spiritual well-being.

Mental health, on the other hand, appears to be affected to a greater extent by spiritual well-being and to a lesser extent by gender. These data are confirmed by previous research which highlighted how spirituality and religious practices are a protective factor and closely connected to physical and mental health (3, 35, 41), as well as being a source of physical and psychological resilience (7, 39) and helping the development of coping strategies in people who experienced stressful life situations (34, 41), mitigating mood disorders such as depression and anxiety (19, 39).

The data presented give us a significant picture of the mental health situation experienced by the Italian population during the first lockdown and confirm the value of spiritual well-being as a protective factor of people's general well-being. However, we would like to outline some limitations of the research. Due to the contextual situation that involved a forced physical distance, the online questionnaire method seemed, despite the limits that this entails, the only possible strategy to reach a large number of subjects. Some limitations of the online questionnaire include the non-completion rate caused by the lower level of engagement than the paper questionnaire and the high number of questionnaires in circulation; moreover, a bias may be dictated by the type of careless response also highlighted in the literature (94). This choice of data collection was also confirmed by other researchers in relation to the COVID-19 epidemic (82). Furthermore, another limit, again related to data collection, is the type of sampling used: a random cascade sampling. Despite the research team's efforts to reach a large and diverse number of participants, use of the online questionnaire may not have allowed the involvement of some target populations. In spite of the weaknesses highlighted, this work has among its strengths the fact of being one of the first research studies conducted on the lockdown period related to COVID-19 in Italy which tries to investigate the role of spiritual well-being and its effects on individual well-being in the population with a multidisciplinary approach. This distance also led to a change in the outlook of some disciplines with a predominantly ethnographic approach, to try to get closer to the issues dealt with in a period such as that of the lockdown in which it was important that research continued to have its role of investigating and seeking knowledge despite non-essential activities having been stopped.

## Conclusion

During the COVID-19 pandemic, people and families experienced a new and sudden situation that forced them to stay indoors for a long period of time. In this context, many people and their families experienced situations of great discomfort, stress and fear related not only to the fear of contagion but also to the economic difficulties for those who saw their income reduced due to the closure of production activities or who experienced situations of loneliness, isolation or conflicts within the home. The pandemic has profoundly changed the way of life in society, starting from the need for “social distancing” even among close relatives (87).

In this context it is very interesting to investigate if and how spirituality has been a form of emotional and psychological support useful for dealing with the loss and anguish of critical moments in life like this. This appears particularly dramatic for the families of those who died in hospital without being able to have their loved ones near them and without the latter being able to celebrate the funeral rites. The data from our study show that in the period of the lockdown those who were able to count on important forms of religiosity and spirituality drew mental well-being from these beliefs. Spirituality helped them to make sense of what was happening and not to feel lost in the face of the radical change in the way of living and conducting social relationships.

Loss, grief, mental health, and spiritual well-being emerge as familiar themes in the lives of many individuals, families, and communities in different contexts. According to Zhai and Du (13) recognizing these individual experiences can enable healthcare professionals to develop personalized strategies to facilitate better adaptation to the situation and therefore promote mental health.

If looked at from the point of view of clinical practice, therefore, it can be seen that dealing with spirituality becomes fundamental; in fact, this aspect needs to be considered to really provide adequate support to individuals, especially those who manifest themselves as strong and solid.

In this regard, the consideration of spiritual needs is necessary to provide a holistic and people-centered intervention (95). By spiritual needs we mean everything that refers to the need to find meaning, value in one's life, peace and a sense of connection. These needs are not necessarily exclusively religious, even those who do not have a religious faith still refer to belief systems that provide feelings of meaning and purpose (46) which in this period of the COVID-19 pandemic seem to assume a role and an even deeper meaning in relation to the bewilderment that people are confronted with in the face of such a pervasive, disruptive event creating fragility, fear and daily uncertainties.

In a certain sense it is precisely at the moment of greatest difficulty that the need for support in spiritual terms becomes stronger, in the hope of finding comfort in one's faith and beliefs. Very often, however, we are faced with inadequate preparation in responding to this type of need (96). In fact, the importance of training health professionals so that they possess the skills to identify and support the spiritual discomfort of patients is increasingly evident (97). A distress which can lead to suffering, a state of anguish due to not being able to feel meaning in life in particular adverse moments, which in some way undermines personal identity (47, 48). Addressing psychosocial and spiritual needs can really contribute to the improvement (98) in the quality of life and well-being of individuals, especially at a time like the one the whole world is now facing and in which diagnostic and medical certainties become increasingly unsure and unconsolidated.

## Data Availability Statement

The raw data supporting the conclusions of this article will be made available by the authors, without undue reservation.

## Ethics Statement

The studies involving human participants were reviewed and approved by Department of Education Sciences of the University of Genoa. The participants provided their written informed consent to participate in this study.

## Author Contributions

IC, NR, RP, and FL conceived the original idea of the study and supervised the findings of this work. NR and IC contributed to data processing and analysis and all wrote and organized the manuscript, in particular NR. IC developed the introduction, the first, and second paragraph. RP and FL wrote the third and four paragraph. While NR and IC presented the methodology, procedure, and data section. All authors discussed the results, presented the conclusion and writer reviewed the document, and approved the final version for submission.

## Conflict of Interest

The authors declare that the research was conducted in the absence of any commercial or financial relationships that could be construed as a potential conflict of interest.
